# Une luxation rare de la hanche post traumatique: antérieure type obturatrice

**DOI:** 10.11604/pamj.2016.24.122.8791

**Published:** 2016-06-08

**Authors:** Rateb Kochbati, Marouene Jlailia

**Affiliations:** 1 Service de Chirurgie Orthopédique et Traumatologique, Institut Kassab d'Orthopédie, Tunis, Tunisie

**Keywords:** Hanche, luxation antérieure, fracture, Anterior dislocation, hip, obturator foramen, hip fracture

## Image en medicine

Les luxations coxo-fémorales traumatiques de l'adulte sont définies par un déplacement permanent postérieur ou antérieur de la tête fémorale hors de la cavité acétabulaire. Parmi ces luxations antérieures, se distinguent le type A, supérieur ou pubien et le type B, inférieur ou obturateur. La variété obturatrice qui survient lors d'un mouvement de flexion, abduction et rotation externe forcées est peu connue. Nous rapportons l'image d'un patient âgé de 19 ans victime d'un accident de la voie publique qui a présenté, suite à une chute d'une moto le membre inférieur en abduction et le genou fléchi, un traumatisme fermé non compliqué de la hanche. L'examen clinique a mis en évidence une impotence fonctionnelle totale de la hanche gauche et une attitude vicieuse en flexion-abduction-rotation externe. Le Bilan radiologique a montré une luxation antérieure type obturatrice. Une réduction de la hanche par des manœuvres douces a été faite sous anesthésie générale. Les suites post réductionnelles étaient bonnes. Les luxations obturatrices traumatiques ont fait l'objet de très peu de travaux. Le pourcentage de luxations obturatrices rapporté dans les séries de luxations traumatiques est compris entre 6 et 10%. Ces luxations doivent être bien identifiées en raison du risque fracturaire lors de la manœuvre de réduction. Les modalités de réduction sont discutées. En absence de lésions associées et de fracture iatrogène lors des tentatives de réduction, le pronostic semble favorable et serait meilleur que celui d'une luxation postérieure.

**Figure 1:**
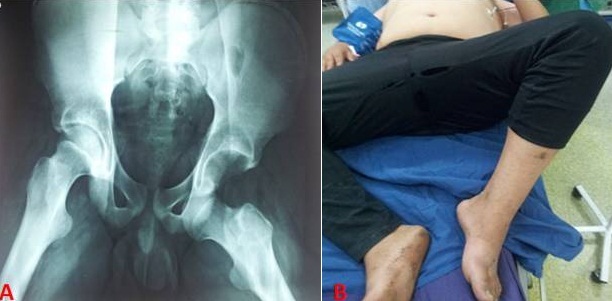
A) luxation antérieure type obturatrice de la hanche gauche; B) attitude du membre inférieur gauche en flexion, abduction et rotation externe

